# Confronted with the real world: how final-year medical students experience interprofessional collaboration in clinical practice

**DOI:** 10.1186/s12909-026-09479-y

**Published:** 2026-05-20

**Authors:** Marwa Schumann, Sophie Michels, Anne Franz, Miriam Alexander, Harm Peters

**Affiliations:** Dieter Scheffner Center for Medical Education and Educational Research Dean’s Office for Study Affairs, Charité – Universitätsmedizin Berlin, Charitéplatz 1, Berlin, 10117 Germany

**Keywords:** Interprofessional education IPE, Final pre-clerkship year, Qualitative study

## Abstract

**Background:**

Interprofessional education (IPE) is increasingly embedded in undergraduate medical curricula, yet little is known about how students encounter interprofessional collaboration (IPC) in real-world clinical practice.

**Methods:**

This longitudinal qualitative study analyzed written diaries from eight final-year medical students at one institution (blinded) to explore IPC experiences during clerkships without formal IPE. Deductive coding used an interprofessional learning framework with four domains: “Roles, Hierarchy, and Power Dynamics,” “Team Inclusion, Collaboration, and Silos,” “Communication Patterns,” and “Navigation of Conflicts.”

**Results:**

Students reported contrasting experiences with professional roles and power dynamics. Supportive environments with clear delegation facilitated learning and participation, whereas rigid hierarchies fostered hostility. Formal communication channels promoted coordination, while informal interactions fostered inclusion and trust. Conflict was infrequent and generally avoided.

**Conclusions:**

These real-world accounts illustrate how traditional cultural norms and local team dynamics shape medical students’ interprofessional interactions in ways that are difficult to reproduce in classroom or simulation settings. To bridge this gap, IPE should be extended into authentic workplace learning as a socially situated process influenced by local practices and expectations. Targeted strategies to strengthen team communication and clarify roles are particularly needed when these elements are weak and especially during transitional phases such as clerkships.

## Introduction

Interprofessional collaboration (IPC) is increasingly recognized as essential for addressing the evolving and often complex demands of modern healthcare systems, particularly in the pursuit of effective, safe, and patient-centered care [1–3]. In response, interprofessional education (IPE) has been incorporated into the curricula of health profession education programs worldwide with the aim to better prepare future healthcare professionals for collaborative practice [4]. Common IPE approaches include various interprofessional small-group formats and integrated teaching modules, which are typically delivered in structured classroom settings [5, 6]. While these approaches offer valuable learning opportunities and preparation for IPC, little is known about how students actually perceive and engage with IPC in authentic clinical workplaces, particularly in the absence of structured interprofessional education [7–10]. This gap may become especially pertinent during final-year clinical placements, when students take on greater responsibilities and begin to transition into professional roles within multiprofessional healthcare teams. The purpose of this study is to explore how final-year medical students experience and interpret interprofessional collaboration during their clinical placements.

The need for IPE to better prepare healthcare students for collaboration in today’s and tomorrow’s complex healthcare environments has been widely recognized by national and international organizations [9, 11]. For example, the Framework for Action on Interprofessional Education and Collaborative Practice by the World Health Organization (WHO) explicitly positions IPE as a foundational strategy for developing a workforce capable of working effectively across professional boundaries [12]. In addition, organizations such as the Interprofessional Education Collaborative (IPEC) in North America, the Centre for the Advancement of Interprofessional Education (CAIPE) in the United Kingdom, and the Committee on Interprofessional Education of the German Medical Association have played prominent roles in actively promoting IPE. They have contributed to the development of national frameworks and core competencies designed to prepare students for the collaborative, interprofessional practice in increasingly demanding, multi-professional healthcare systems [13–16].

In response to the growing emphasis on interprofessional collaboration, various formats of IPE have been introduced into the curricula of many health professions education programs worldwide, although often to a limited extent and with varying degrees of integration [17, 18]. Commonly, IPE is delivered in structured classroom settings and ranges from isolated interprofessional small-group sessions to more comprehensive, integrated IPE modules [19, 20]. Frequently used formats for IPE include, for instance, problem-based learning, case-based seminars and lectures, and simulation-based education [5, 6]. These approaches often take place in educational environments where, to some extent, a shared understanding and conceptual framework for interprofessional collaboration has already been established. However, this structured and somewhat idealized context may reduce students’ perception of the authenticity and relevance of IPE [21]. As a result, many students complete their undergraduate studies with limited preparation for the complex and dynamic interprofessional interactions they will encounter in real-world clinical practice [22, 23].

In contrast to the role of structured IPE in preparing healthcare students for interprofessional collaboration (IPC), comparatively little attention has been paid to how students actually encounter and engage with IPC in authentic clinical settings [24]. Workplace learning in health professions education differs significantly from classroom-based learning. It is multifaceted, encompassing formal instruction, informal interactions, elements of the hidden curriculum, and socialization processes through which learners internalize professional norms, hierarchies, and collaborative behaviors. This gap can become especially relevant during final-year clinical placements, when students assume increasing responsibility for patient care and begin transitioning into professional roles within multiprofessional healthcare teams. Exploring this under-researched dimension can offer important insights into how students learn to collaborate across professional boundaries in the absence of formal instruction. Addressing this gap may contribute to the development of educational strategies that more accurately reflect the realities of clinical practice and better support students for IPC and in becoming effective members of interprofessional teams.

The aim of this study is to explore how final-year medical students experience and interpret interprofessional collaboration in clinical workplace settings. Using a qualitative approach, the study draws on students’ reflective diary entries to illuminate how they perceive, navigate, and learn from interprofessional encounters in authentic clinical environments where no structured interprofessional education is in place.

## Methods

### Philosophical assumptions and study design

A qualitative design was chosen to explore the interprofessional experiences of medical students during their final year of clerkship based on online diaries completed by individual students. A hermeneutic stance underpins this study within the broader framework of constructivist theory [25]. This perspective is particularly suited to the qualitative exploration and interpretation of complex social interactions, recognizing that the experiences under investigation are not simply documented by the data, but are also co-constructed, incorporating both the perspectives of the researchers and the potential for multiple interpretations of reality [26].

### Study setting

This study was conducted at the Charité Universitätsmedizin-Berlin, where the undergraduate medical program extends over six years and follows an integrated, competency-based curriculum with a modular structure (Modular Curriculum of Medicine) [27]. It combines theoretical, preclinical and clinical content in structured learning modules, with students attending clinical courses from the first semester.

The final clerkship year consists of 48 working weeks, divided into three rotations of 16 weeks each: one in internal medicine, one in surgery and one in an elective discipline [28, 29]. There is no formal interprofessional curriculum at this stage. Clerkships take place either on the Charité campus or in affiliated teaching hospitals, with some students completing rotations at other medical universities in Germany. The primary goal of the final year of clerkships is to provide students with hands-on experience in patient care under real clinical conditions and to prepare them for their role as physicians after graduation. Under the supervision of experienced physicians, students gradually take on more responsibility and gain increasing autonomy in performing clinical tasks [25].

### Data collection

Final year clerkship students at the Charité were invited to participate in the study through announcements on the institution’s website and wall posters. Participation was voluntary, and students provided written informed consent prior to data collection. As part of their participation, participants received financial compensation for up to 10 h of work per month. In addition to mandatory rotations in internal medicine and surgery, students had the opportunity to complete elective rotations in fields such as anaesthesiology, neurology, ophthalmology, radiology, and urology. A purposive, maximum variation sampling strategy was used for this study to capture a wide range of experiences and increase data richness [30].

### Qualitative data analysis

Data collection and analysis took place simultaneously in an iterative process. Qualitative content analysis approaches vary according to the degree of inductive reasoning [31, 32]. For this study, a directed qualitative content analysis was used, where diary data entries were analyzed deductively based on a pre-existing framework, in our case the conceptual framework of interprofessional learning outcomes (ILO) (as opposed to conventional qualitative content analysis, where coding categories are derived directly and inductively from the raw data, often used in grounded theory) [33]. Coding was guided by the ILO framework to provide a structured lens through which to examine the data. This iterative process of analysis ensured that while the theoretical framework guided the interpretation, the findings remained grounded in the participants’ experiences [34].

Following the stages of qualitative content analysis, the author SM began with decontextualization by breaking down the diary entries into smaller units and coding key phrases or concepts relevant to the research question, based on five diaries, after which data saturation was reached [35]. Recontextualization was then undertaken by authors MS, AF, MA and HP, who reviewed the initial codes to ensure they captured the data accurately while maintaining context. In the categorisation phase, the same authors grouped similar codes into themes to reveal patterns within the data. Finally, in the synthesis phase, they explored the relationships between these themes to draw conclusions and insights. Throughout the process, the codes were iteratively revised and modified to accurately reflect the data [30]. Throughout each stage, all authors consistently reviewed coding and interpreting data. Any discrepancies in interpretation were resolved collaboratively through discussion, negotiation and, ultimately, consensus among the entire team of authors [36]. The quotes presented in this article were translated by the author HP. In addition, translated excerpts were reviewed within the research team to ensure that meanings were preserved as closely as possible. Diary analysis was carried out using MAXQDA software.

### Ethical approval

This study was conducted in accordance with the ethical principles of the Declaration of Helsinki. Ethical approval for the study was granted by the Charité data protection office (No. AZ 721/16) and the Charité ethics committee (No. EA1/342/20). Written informed consent was obtained from all participating final clerkship students, participation was entirely voluntary with no coercion involved, and all data were anonymized to ensure confidentiality.

### Quality, rigor and reflexivity

Several methods were used to ensure the quality and rigor of the current study. Credibility was ensured by triangulation of researchers, with data analyzed by more than one person, and by the inclusion of more than one location, within and outside of the Charité, to ensure comprehensiveness and more reflexive data analysis [37]. Validity was achieved by constructively aligning the research question, the constructive epistemology underpinning the research, and the qualitative method used [38].

In order to maintain the quality of research, reflexivity about the relationship between researchers and participants is crucial [39]. In this study, we adopted an outsider’s perspective - one not rooted in direct experience of the professional settings in which final-year clerkship students at the Charité operate. This position had the advantage of facilitating an objective coding and analysis process, minimising the influence of personal bias. However, it also posed challenges, particularly in fully capturing the nuanced dynamics of interprofessional encounters and in establishing a deep rapport with the participants, who detailed their experiences in written diaries. Member checking was not feasible, as participants were no longer reachable after graduation and completion of data collection.

Given the diverse professional, national and cultural backgrounds of our interdisciplinary research team - including expertise in medicine, medical education, sociology, psychology and adult education - and our varying levels of experience with qualitative research, we continually reflected on how our outsider status might affect the interpretation of the data. This reflexivity was crucial in ensuring that our analysis - structured around themes such as roles, hierarchy, communication patterns and conflict navigation - remained both comprehensive and sensitive to the complex realities of interprofessional practice. Ultimately, this approach enriched our findings and provided robust insights into how leadership, team dynamics and institutional culture shape the interprofessional experiences of medical students during their final year of clerkship.

## Results

### Participants

Eight final-year clerkship students (six women, two men) aged between 25 and 28 years and distributed across nine different training sites in and outside the Charité participated in the study. The most prominent interprofessional interactions described across the diary entries involved physicians and nursing staff. Encounters with other healthcare professionals, such as physiotherapists, dieticians, diabetes assistants, social workers, psycho-oncologists and administrative staff, were also reported, but these were not the primary focus of participants’ accounts.

### Coding Framework

**Fig. 1:**
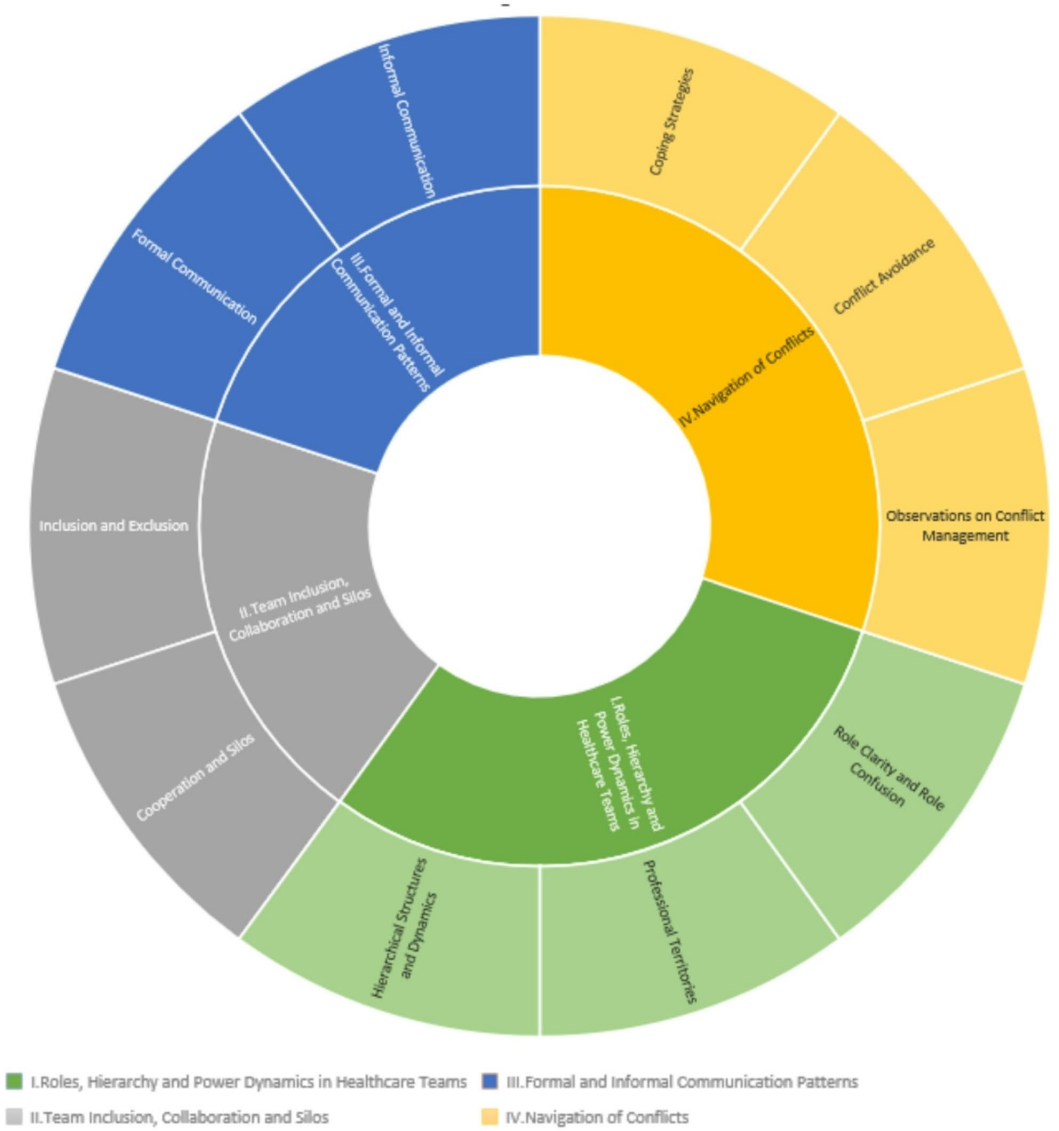
Coding framework

The original framework by Behrend et al. provided a structured approach to analyzing interprofessional teamwork, focusing on key aspects such as roles, collaboration, communication and conflict management [33]. We adapted this framework to better capture the complexity of our participants’ experiences by refining the themes to emphasize hierarchy and power dynamics, team inclusion, communication patterns, and conflict navigation, allowing for a more nuanced analysis of workplace interactions (Table 1; Fig. 1). The quotes presented below are illustrative examples, selected to represent recurring patterns identified across the dataset.

**Table 1:** Definitions of themes

Theme	Definition
Roles, Hierarchy, and Power Dynamics	Explores how medical students experience and navigate professional roles, hierarchies, and power structures in healthcare teams.
Team Inclusion, Collaboration, and Silos	Explores interprofessional collaboration in healthcare settings and the inclusion/exclusion of students in clinical teams.
Formal and Informal Communication Patterns	Analyzes communication dynamics in interprofessional teams, examining both structured workplace communication and informal interactions.
Navigation of Conflicts	Investigates how medical students observe, experience, and manage conflicts in interprofessional healthcare teams.

### Roles, hierarchy and power dynamics

Analysis of this theme revealed a wide range of experiences, from supportive to hostile interactions, and from clear, well-organized environments with explicit role delegation to confusing situations where role ambiguity created tensions that significantly affected both the learning and team functioning. Subthemes included hierarchical structures and dynamics, professional territories, and role clarity/confusion.

Analysis of the subtheme “Hierarchical Structures and Dynamics” showed both overt and subtle manifestations of hierarchical relationships with members of the interprofessional healthcare teams. Collegial and collaborative dynamics emerged when personal factors such as age, reduce the perceived distance between participants and members of the healthcare team (Table 2, quote 1). Power was asserted through both direct actions and implicit communicative practices, e.g. assigning unnecessary tasks as a means to reinforce hierarchical control by nurses (Table 2, quote 2). Formal patterns of communication contributed to the maintenance of these hierarchies, highlighting the rigid, top-down flow of communication that may limit interprofessional dialogue (Table 2, quote 3). Language emerged as a subtle mechanism for reinforcing power dynamics through the contrast in pronouns between the formal ‘you’ (Sie), signifying respect and social distance, and the informal ‘you’ (du), signifying familiarity and equality. This explicit linguistic structure not only reflects, but also perpetuates established professional boundaries, signaling to participants the implicit hierarchical rules they must internalize to successfully navigate clinical settings (Table 2, quote 4). As a result, certain individual behaviors can reinforce hierarchical attitudes and exacerbate tensions within teams (Table 2, quote 5).

The analysis of the sub-theme “Professional Territories” revealed that ambiguous task sharing creates tension and uncertainty about task boundaries, sometimes complicating the learning process (Table 2, quote 6). Similarly, territorial behavior can be interpreted as a form of power assertion, with students often the targets of such overt displays of authority, which can impair the cohesion of interprofessional clinical teams (Table 2, quote 7).

Analysis of the sub-theme “Role Clarity and Role Confusion” revealed that the degree of clarity of role definitions within healthcare teams has a significant impact on interprofessional collaboration and participants’ experiences. Clear role definitions and effective task delegation are essential for maintaining effective interprofessional collaboration (Table 2, quote 8). In contrast, unclear roles contribute to practice challenges, increased tension and negative emotional impacts (Table 2, quote 9). Similarly, multiple accounts of overlapping responsibilities between nursing and medical staff led to interprofessional tension, creating a struggle for participants to navigate overlapping roles (Table 2, quotes 10 and 11).

In summary, professional roles and hierarchies during the final year of clerkship training ranged from positive, supportive environments where hierarchies were constructively managed and roles were clear, to challenging, hostile environments characterized by aggressive hierarchical behavior, territorial conflicts and role confusion. The contrasts in participants’ experiences suggest that positive experiences often depend on individual leadership and team dynamics rather than systematic approaches to interprofessional collaboration.

**Table 2:** Roles, hierarchy and power dynamics

Nr.	Quote	Participant
1	“The age difference between her (the nurse) and me wasn’t particularly large, which created a different level of collaboration through the smaller distance that usually results from the product of age and hierarchy.”	Male participant, Anaesthesiology
2	“Today a nurse yelled at me again, telling me to clean the blood collection tray. I think she just wanted to show me the hierarchy, because it was already clean.”	Female participant, Surgery and internal medicine
3	“The relationship between doctors and nurses is quite formal. I noticed that the senior doctor usually only communicates with the head nurse, interactions with other nursing staff are rare and mainly consist of giving orders.”	Female participant, internal medicine
4	“Doctors expect to be addressed formally (Sie), while nurses are addressed informally (du).”	Female participant, surgery, Anaesthesiology
5	“The surgeon’s hierarchical attitude caused unnecessary tension in the operating room.”	Male participant, Anaesthesiology
6	“Sometimes I feel that the anesthesia nurses resent me for taking over some of their duties. This has a lot to do with the fact that the division of work between the anesthesiologist and the nurse anesthetist is not clear or defined enough”.	Male participant, Anaesthesiology
7	“The nurses wanted to show me that they’re the bosses on this ward. They kept sending me to the lab with blood samples, constantly telling me to clean the blood collection tray, and so on.”	Female participant, internal medicine and surgery
8	“A structured task delegation by the chief anesthetist prevented chaos…. The tasks were well-delegated by the senior physician, which made the situation manageable.”	Male participant, Anaesthesiology
9	“It wasn’t clear who was supposed to do the blood draws, which led to frustration.”	Male participant, Anaesthesiology
10	“Role overlaps caused confusion between nursing and medical duties.”	Male participant, Anaesthesiology
11	“The overlap of responsibilities between doctors and nurses caused tension.”	Female participant, internal medicine

### Team inclusion, collaboration and silos

Analysis of this theme revealed the complex dynamics of interprofessional collaboration during the final year of clerkship. Diary entries reveal contrasting experiences, from feeling valued as a team member to feeling like a complete outsider. Subthemes included inclusion/exclusion and cooperation/ silos.

The sub-theme of “Inclusion/Exclusion” included significant contrasts in participants’ experiences of being included or excluded from health care teams. For some participants, small acts of recognition fostered a strong sense of belonging (Table 3, quotes 1 and 2). However, the variability of cultures across specialties and departments has contributed to inconsistent experiences where constant team changes require a high level of adaptability (Table 3 quotes 3 and 4). In contrast, experiences of exclusion had the negative emotional impact of not only diminishing a participant’s sense of belonging, but also hindering their overall learning and well-being (Table 3, quote 5).

**Table 3:** Team inclusion, collaboration and silos

Nr.	Quote	Participant
1	“Small gestures like being called by name made me feel part of the group. All the doctors and nurses make me feel that I am an integral part of the team. That feels good and motivates me to work.”	Male participant, Anaesthesiology
2	“Feeling like a valued team member encouraged me to take more initiative.”	Male participant, internal medicine, neurology
3	“Some wards were more welcoming than others, which affected my overall experience”	Male participant, Anaesthesiology
4	“During my 6-month final clerkship training so far, I have worked in four different departments (…) and had to learn to integrate into new teams each time and work on being accepted there. Successfully mastering this is a great challenge and good experience for my later professional career, in addition to the new medical knowledge!’	Internal medicine, neurology
5	“I felt out of place when team members ignored my presence (…) made me feel isolated. “Nobody was interested in whether I needed help or not. But unfortunately, that’s how it often is as a final year student and later as a junior doctor - you have to teach yourself everything because no one else will show you.”	Female participant, internal medicine
6	“The nurses seem nice but aren’t actually nice. They wanted to show the young resident physicians that they have the power here. I’ll be glad when I don’t have to work with this team anymore.”	Female participant, surgery and internal medicine
7	“The co-operation with the nurses worked well, they were always happy when I placed flex tubes immediately, I was happy when they always told me in good time where to place a flex tube. (…) I could always ask questions and was always told where there was something for me to do (placing venous cannula, blood sampling, patients’ admissions). I was happy to take on these tasks.’	Female participant, internal medicine
8	‘Everyone on the ICU works closely and constructively together. Many nursing staff here have a wealth of experience. We benefit from each other. (…) You can really feel how much the intensive experiences of an intensive care unit weld us together. We all pull together.’	Male participant, Anaesthesiology
9	“There is a lot of time pressure in the emergency department. As a physician, you are directly dependent on the preparatory work of the nursing staff (measuring vital signs, ECG, inserting venous access, taking blood samples) and have to make decisions much more quickly than on the ward. On the other hand, I also enjoy working closely with the nursing staff.”	Male participant, internal medicine, neurology
10	“Doctors and nurses eat in separate rooms, limiting interaction.”	Male participant, Anaesthesiology
11	“The atmosphere on the ward is not good at all. People don’t talk to each other during breakfast. Even the seating at meals is divided between medical and nursing staff. (…) There is relatively little contact between nursing and doctors on this ward. Contact is limited (as far as I can tell) to medical orders and the most necessary exchange of information.”	Male participant, internal medicine, neurology
12	“Separate workflows created unnecessary barriers to teamwork. (…) The team was divided along professional lines, which affected cohesion.”	Female participant, internal medicine

Analysis revealed a clear link between the subthemes inclusion/exclusion and hierarchical structures and dynamics where some participants felt excluded through hierarchical authority and interpreted seemingly polite behaviour as a reinforcement of power imbalances. Thus, the degree of inclusion is closely linked to these underlying hierarchical structures, which, in turn, have a significant impact on their learning and professional identity formation (Table 3, quote 6).

Analysis of the “Collaboration and Silos” subtheme revealed that interprofessional collaboration can be facilitated or hindered by organizational structure and physical separation. On the positive side, several participants described effective interprofessional collaboration and supportive dynamics that not only facilitated the performance of essential clinical tasks, but also fostered a strong sense of interprofessional teamwork and mutual respect (Table 3, quote 7). Especially in the ICU and emergency department, shared challenges and recognition of interdependence and expertise can foster positive interprofessional relationships and a cohesive team environment (Table 3, quotes 8 and 9). On the other hand, the organizational structure and physical separation of different professions may limit interprofessional interaction (Table 3, quotes 10 and 11). In addition, organizational factors such as separated workflows and rigid professional boundaries created fragmented communication channels and unnecessary barriers to interprofessional teamwork (Table 3, quote 12).

In summary, some healthcare environments foster effective interprofessional collaboration through shared experiences, inclusive cultures, and interdependent workflows, while others remain rooted in rigid organizational silos and physical separations that limit interprofessional teamwork. Addressing these educational challenges is essential not only for improving student learning and professional development, but also for improving overall patient care in increasingly complex healthcare systems.

### Communication patterns

Analysis of this theme reveals how participants observe and experience interprofessional communication in clinical settings, and how communication dynamics can either foster collaboration or create divisions within healthcare teams. In line with the focus of this study, communication is understood as occurring within a broader interprofessional team context, where both intra- and interprofessional interactions shape collaborative practice. Subthemes include formal and informal communication.

Analysis of the sub-theme of ‘Formal Communication’ shows an overall positive experience for participants in structured clinical settings, particularly in high-pressure emergency where effective communication prevented chaos and ensured coordinated action (Table 4, quote 1). Similarly, effective formal communication emphasizes shared responsibility for patient care (Table 4, quote 2). Although this example reflects communication between two medical specialties, it illustrates coordination processes within the broader interprofessional team. From the perspective of the study participants, such interactions are experienced as part of a shared team environment in which communication patterns and hierarchies influence interprofessional collaboration. In other situations, however, formal communication was perceived as rigid, hierarchical or inadequate, limiting effective information sharing and interprofessional collaboration, i.e. strictly task-oriented communication between professions (Table 4, quote 3). Language played an important role in shaping the communication, particularly in relation to professional hierarchies. In German, the distinction between the informal first name basis and the formal surname basis is rooted in workplace culture and serves as a marker of status and authority. Some participants observed traditional hierarchies in which nurses are addressed informally, suggesting a lower professional status, while senior doctors are addressed formally, reinforcing their authority (Table 4, quote 4). In addition, the challenge of obtaining essential details about patient care highlights barriers to effective communication where participants have to actively seek out information rather than being included in formal knowledge sharing processes (Table 4, quote 5).

**Table 4:** Communication patterns

Nr.	Quote	Participant
1	“During the emergency situation, I was impressed by how the chief physician took charge, assigned clear tasks so that everyone who rushed in had a role and no chaos broke out.”	Male participant, Anaesthesiology
2	“Very pleasant working atmosphere. The anesthetist and surgeon communicate very intensively. It is clear that this is a ‘joint’ patient.”	Male participant, Anaesthesiology
3	“Communication between professions was minimal and strictly task-oriented.”	Male participant, internal medicine, neurology
4	“I’m always struck by how old-fashioned surgery can be. Nurses are always addressed on an informal first-name basis, but senior physicians and the department heads expect you to address them on a formal surname basis…”	Female participant, surgery, anaesthesiology
5	“To get information about examinations, you have to be very persistent and ask questions multiple times.”	Female participant, internal medicine
6	“I find it very pleasant that the doctors in the intensive care unit have breakfast together in the meeting room. Information is exchanged in a relaxed atmosphere, and you get the impression that all the physicians start the day more motivated. I have also found the interaction between physicians and nursing staff to be much more harmonious here than in the operating theatre. They know and appreciate each other. They addressed each other almost everywhere on an informal first-name basis.”	Male participant, Anaesthesiology
7	“And when a young junior doctor also chats to me about personal matters, I feel accepted and happy.”	Female participant, internal medicine
8	“Close cooperation with the anaesthetist and the anaesthesia nurse. A very nice working relationship has developed with her. She calls me by my first name and we sometimes chat about things outside of work during the break.”	Male participant, Anaesthesiology
9	“At breakfast I find the atmosphere strange. Everyone has their designated seat and the doctors don’t talk with the nurses. Only when they need to discuss patients.”	Female participant, surgery and internal medicine

Analysis of the “Informal Communication” sub-theme shows how casual, non-task-oriented interactions fostered a sense of inclusion, motivation and collaboration, helped bridge hierarchical gaps and facilitated teamwork. Several participants described positive experiences with informal communication, particularly in settings where interprofessional relationships were more collegial, such as how shared morning breakfasts and the use of first names contributed to a relaxed and supportive atmosphere that enhanced teamwork and motivation (Table 4, quote 6). Similarly, informal conversations played a key role in participants’ sense of belonging and acceptance within the team, e.g. how engaging in personal conversations contributed to feeling included (Table 4, quote 7).

The role of language is again evident, with the use of first names and casual conversations appearing to contribute to a sense of collegiality and trust, reinforcing the importance of informal interactions in building effective interprofessional relationships (Table 4, quote 8). However, the lack of informal communication was also noted in other settings, contributing to a distant atmosphere where interprofessional interaction was strictly limited to professional necessity: Table 4, quote 9).

In summary, the analysis of communication patterns in clinical settings highlights the dual impact of formal and informal communication on interprofessional collaboration, team integration and workplace dynamics. While structured communication ensures clarity and efficiency, informal exchanges contribute to a positive work culture, enhance team dynamics, and provide valuable learning opportunities for medical students.

### Navigation of conflicts

Analysis of this theme reveals how participants perceive, experience, and manage conflict within interprofessional health care teams. Overall, the participants’ reports of their clinical experiences were relatively positive, and direct involvement in conflict was rare. Most conflict-related diary entries described situations that participants observed rather than those in which they were actively involved. Overall, students frequently relied on avoidance as their primary strategy for managing workplace conflicts, often out of concern for potential negative consequences. Subthemes included conflict management approaches, conflict avoidance and coping strategies.

Analysis of the sub-theme of “Conflict management approaches” revealed a wide range of conflict resolution strategies among healthcare professionals described by participants, including both constructive and dysfunctional approaches to conflict management. In instances of effective conflict resolution, participants described healthcare professionals who handled disagreements in a professional and calm manner fostering a positive and collaborative atmosphere (Table 5, quote 1). Conversely, participants also encountered dysfunctional conflict management where negative behaviours contributed to a stressful and counterproductive work environment (Table 5, quote 2). In more escalated conflicts, participants witnessed unprofessional behaviour and verbal violence, including yelling and insults at both nurses and students, an interaction that was visibly disapproved of by other team members (Table 5, quote 3). In addition, some participants described work environments where chronic stress and unresolved tensions led to ongoing negative interactions, characterized by rushed communication and visible frustration among healthcare providers (Table 5, quote 4).

**Table 5:** Navigation of conflicts

Nr.	Quote	Participant
1	“The nursing staff and the ward physician worked well together today, the nursing staff only pushed a little during the ward round and asked for a slightly quicker round, but the ward physician took this very calmly and clarified it with the nursing staff in a friendly manner. (….) I could tell that the male nurse was a bit annoyed by the length of the ward round (until 1 pm), but he was always polite and friendly, which I liked.”	Female participant, internal medicine
2	‘The surgeon, …, reacted very indignantly to every exchange of words, to instruments being incorrectly loaded by the theatre nurse, etc. It was interesting to see how this led to a bad atmosphere, which certainly encouraged further errors, especially on the part of the nursing staff.’	Male participant, anesthesiology
3	‘The head physician for anaesthesia showed completely unacceptable behaviour towards the nursing staff and also towards a PJ student who was present. He was shouting and insulting. Too bad, … I observed a lot of head shaking.’	Male participant, anesthesiology
4	“The interaction was as usual - everyone was annoyed and rushed. Stressed responses and unfriendly faces. “	Female participant, internal medicine and surgery
5	“Otherwise, interaction with the nurses is limited to the bare necessities. I try to avoid them as much as possible. (….) I try to stay out of their way as much as possible”	Female participant, internal medicine
6	“The nurses in the emergency department are very well trained and competent. Nevertheless, under stress, they tend to attack other (young) junior doctors or final clerkship students in particular and tell them off (‘No, doctor! That’s not how it works! I need this room, you can’t go in there!’) … Each time I waver between keeping quiet and giving in, or contradicting and refuting the accusations. Unfortunately, I know that one can only draw the short straw. Because when the nurses don’t like you, you don’t get shown or explained anything anymore. Then you’re ignored.”	Female participant, internal medicine
7	“The senior physician is often very irritable and stressed and makes silly comments, but you quickly learn not to take it personally and just half-listen.”	Female participant, surgery, anesthesiology
8	“I learned that one has to integrate into new teams each time and work on being accepted there.”	Male participant, internal medicine, neurology

Analysis of the “Conflict Avoidance” sub-theme revealed that participants often engaged in avoidance behaviors when faced with workplace tensions. Rather than actively engaging in conflict resolution, many participants chose to minimize interactions with certain team members to avoid potential confrontation, which in turn limited their learning opportunities and professional integration (Table 5, quote 5). In other situations of power imbalance, where standing up for oneself could result in being ignored or excluded from educational opportunities, participants face the dilemma of either defending themselves or giving in to perceived unfair treatment, ultimately concluding that avoidance is the safest approach (Table 5, quote 6).

Analysis of the “Coping Strategies” sub-theme revealed a variety of informal approaches to dealing with workplace conflict, e.g. emotional distancing as a means of shielding oneself from workplace tensions, to cope with the irritable and dismissive behavior of a senior physician (Table 5, quote 7). In contrast, other participants described a more active and constructive approach, recognizing the need to integrate into new teams and foster acceptance, reflecting an awareness that successful team integration requires effort and adaptability (Table 5, quote 8). While this approach encourages collaboration, it also implies that participants perceive the burden of adaptation as falling primarily on them, rather than being supported by a structured educational framework.

In summary, participants primarily observed rather than engaged in workplace conflict within interprofessional health care teams, often relying on avoidance to manage tensions due to concerns about negative consequences. Approaches to conflict management varied from effective resolution strategies to a stressful and unprofessional environment. Coping strategies ranged from emotional distancing to proactive team integration, although participants felt that the burden of adjustment was largely on them, rather than being supported by a structured framework.

## Discussion

Interprofessional collaboration is an essential competence for healthcare professionals, yet little is known about how medical students experience it in authentic clinical environments. This study focuses on final-year medical students during their clerkship year and explores their experiences of IPC in the absence of formal IPE. Our findings reveal that students’ engagement in interprofessional practice is influenced by four interconnected domains: the impact of roles, hierarchy and power dynamics on their position within teams; the varying extent to which they are included in and collaborate with teams, the dual effect of formal and informal communication patterns on information flow and relationships; and the prevalence of conflict avoidance as a strategy for navigating workplace tensions. These observations underscore how prevailing cultural expectations and on-the-ground team processes shape students’ interprofessional learning and engagement, effects that are difficult to recreate in classroom or simulation environments. In the following, we will elaborate on and discuss these themes in light of current literature and their implications for medical education.

In terms of roles, hierarchies and power dynamics, our findings showed that final-year medical students entering clinical environments often feel uncertain about their role in the healthcare team, finding themselves caught between the roles of learner and team member. This ambiguous status frequently created uncertainty regarding their authority and responsibilities, echoing previous research in which power imbalances and unclear roles were identified as barriers to professional growth and collaborative learning [40, 41]. Even in contexts where IPE initiatives are in place, hierarchical team structures and professional stereotyping persist, thereby reinforcing an established authority gradient [42–44]. Our study adds to this body of work by highlighting how culturally rooted hierarchical markers—such as the formal versus informal address in the German language—can subtly reinforce professional boundaries, influencing team climate and the quality of interprofessional collaboration [45]. Nevertheless, evidence from recent intervention studies demonstrates that these barriers are not immutable; structured IPE programs and interprofessional simulations have been shown to enhance role clarity, soften authority gradients, and increase trainees’ readiness to collaborate across professional boundaries [1, 7, 40]. The limited translation of these positive outcomes into everyday clinical environments may be explained by findings from curriculum mapping studies, which reveal that while most programs incorporate roles and responsibilities into IPE competency frameworks, this domain is frequently underdeveloped in real-world workplace learning, leaving students confronted with the ‘real world’ of complex team dynamics [46].

Regarding team inclusion, collaboration, and silos, our findings indicate that final-year medical students’ sense of belonging varied widely across clinical environments, ranging from experiences of genuine integration into interprofessional teams to feelings of exclusion and marginalisation—a pattern also observed in studies showing that organizational culture and professional boundaries strongly influence learners’ inclusion [21, 43]. Even in settings with formal IPE exposure, workplace silos and professional boundaries often persist due to entrenched professional mindsets and heterogeneous learning cultures [44, 47]. These findings suggest that interprofessional learning in the workplace is fundamentally different from classroom-based IPE. The authentic, dynamic, and sometimes unpredictable context of clinical work cannot be fully simulated, making on-the-job exposure crucial for developing adaptive competencies. In contrast, other studies have reported consistently high levels of student inclusion when IPE competencies are embedded in longitudinal curricula, interprofessional seminars are reinforced through clinical application, and sustained team-based problem-solving approaches are employed [46, 48]. These interventions, alongside evidence from interprofessional simulation and extended placements, suggest that sustained exposure to inclusive team environments strengthens learners’ ability to collaborate effectively, whereas short or fragmented experiences offer fewer benefits [46, 48]. Our study contributes to the existing literature by demonstrating that the degree of inclusion in everyday clinical placements is influenced not only by organizational structures, but also by the microcultures of individual wards, reinforcing the idea that workplace learning is a socially situated process shaped by local team norms and practices.

Regarding formal and informal communication patterns, our findings indicate that structured, task-oriented exchanges often ensured clarity and efficiency in high-pressure situations, while informal interactions fostered a sense of inclusion, bridged hierarchical gaps, and promoted collaborative teamwork. These observations reflect the broader understanding that both formal and informal communication play critical roles in shaping medical students’ interprofessional experiences, with formal training improving skills and informal interactions fostering understanding and collaboration [49, 50]. Evidence from the literature demonstrates that formal communication is a skill that can be deliberately developed through simulation exercises, workshops, and structured team-based activities, with such formats consistently enhancing students’ confidence, role clarity, and ability to convey information effectively [51]. Creating opportunities to reflect on these experiences—through structured coaching groups or peer discussions—can help students critically process and integrate their observations into professional growth.

Regarding conflict navigation, participants were occasionally directly involved but more often acted as observers or intermediaries, learning vicariously how conflicts were handled. A common sentiment was uncertainty about when and how to speak up, reflecting a conflict avoidance strategy rooted in their junior status. This is consistent with existing literature, which suggests that conflicts within healthcare teams are an inevitable aspect of teamwork in complex environments such as hospitals, and are often suppressed due to power imbalances [40, 52]. Being confronted with these real-world situations highlights the importance of explicit preparation for conflict navigation. Most students reported receiving little formal training in conflict management; instead, they relied on their personal ethics or informal role modelling. Where implemented, IPE should also train students to anticipate and manage less constructive interprofessional encounters, equipping them with practical engagement and reflection strategies, conflict-resolution frameworks and de-escalation techniques [52, 53].

Our findings indicate that acquiring interprofessional competencies is not merely a cognitive process, but rather, it is deeply embedded in the sociocultural context of clinical learning environments. This perspective is consistent with previous work which conceptualises interprofessional learning as occurring within the ‘nexus’ of education and practice. This work emphasises that competencies develop through participation in authentic care delivery systems rather than isolated educational activities [54, 55]. Similarly, guidance from the National Collaborative for Improving the Clinical Learning Environment (NCICLE) highlights the clinical workplace as a critical site for interprofessional learning, where team-based care, patient safety and professional socialisation are co-constructed. These workplace-based experiences also contribute to the development of interprofessional identity as learners negotiate their roles and sense of belonging within multiprofessional teams [56]. The variation in students’ opportunities for meaningful interprofessional engagement, even within the same institution, suggests that curricula alone cannot guarantee the implementation of IPE principles. Learning IPE is fundamentally workplace-based and cannot be fully replicated in simulated settings. This aligns with the sociocultural learning theories, particularly situated learning theory and the concept of communities of practice [21, 57]. According to these frameworks, learning is not merely the acquisition of knowledge; it is a process of becoming shaped by legitimate participation in real-world social contexts. Situated learning theory emphasizes that learning occurs through participation and direct interaction with experienced practitioners. Medical students, especially those in their final year, enter professional communities on the periphery and gradually transition to full participation. This socialization process is shaped by interpersonal interactions, institutional norms, unspoken rules, and power dynamics. Immersing medical students in health systems can positively influence the development of their competencies and professional identity with regard to IPC [21].

### Implications for IPE curriculum

In terms of curriculum development, contextually relevant IPE opportunities should be integrated into workplace-based learning throughout the clinical years rather than being confined to preclinical or simulated settings. Authentic, workplace-based learning enables students to experience the realities of team dynamics and organizational complexities, developing an adaptive competence that cannot be achieved through classroom-based training alone. Crucially, IPE preparation must extend beyond teaching idealized collaboration and explicitly address how to manage suboptimal or negative interprofessional real world encounters. Furthermore, curricula should intentionally create opportunities for structured reflection, such as coaching groups or guided peer sessions, so that students can critically engage with their experiences, including difficult encounters. These approaches recognize that IPC learning is not a discrete, classroom-based event, but an ongoing process of professional socialization within dynamic and unpredictable clinical environments.

### Strengths and limitations

A key strength of this study is its focus on the real-world, authentic workplace experiences of final-year medical students, rather than on evaluating a structured interprofessional education (IPE) intervention, as is common in much of the existing literature. This approach sheds light on the sociocultural and organizational dynamics that influence collaborative practice in everyday clinical settings, a topic that is frequently overlooked in intervention-based research. Methodologically, longitudinal reflective diaries enabled participants to document their experiences over time and capture strong emotional responses, such as anxiety, confusion, and helplessness, that are often underreported in other data collection methods [58]. Diaries have the advantage of identifying demanding clinical situations and relationship dynamics, thereby supporting targeted curriculum development. Additionally, reflective diaries expose the influence of the hidden curriculum, which may remain invisible in survey- or interview-based research [59].

This study has some limitations. First, since the sample of participants was drawn from a single training context—final-year students within one medical education program—the described experiences and perceptions may have been influenced by the particular institutional culture and healthcare setting. Students in different regions or systems may report different challenges in interprofessional collaboration. Secondly, we only captured the perspective of medical students, without including the views of other professional students or qualified practitioners. Future research employing multi-perspective and multi-site designs that include learners and mentors from various professions would provide a more holistic picture of teamwork during clerkships and capture the complex interplay between professional roles, organizational culture, and learner development. Additionally, examining how different structural interventions, such as longitudinal IPE placements, integrated interprofessional assessment, or co-mentorship models, affect the sustainability of collaborative behaviors after graduation is an area for future research. Longitudinal follow-up of medical graduates could clarify whether early experiences of inclusion or exclusion influence subsequent professional conduct, interprofessional attitudes, and career satisfaction.

## Conclusions

In conclusion, our study of final-year medical students’ clerkship experiences reveals how the realities of clinical workplaces shape the interprofessional interactions and influence the professional development of medical students. These findings contribute to research indicating that sociocultural dynamics, more than formal curricula alone, can play a critical role in shaping collaborative practice. By illuminating how students navigate these settings, this study provides an empirical foundation for rethinking the integration of interprofessional learning in undergraduate medical education, particularly by extending it into authentic clinical placements, to better support their IPC learning and practice.

## Data Availability

Data supporting the findings of this study are available from the corresponding author upon reasonable request.

## Abbreviations

IPE: Interprofessional Education
IPC: Interprofessional Collaboration
